# Ovariotomy in a Patient Sixty-five Years of Age, Complete Recovery

**Published:** 1876-09

**Authors:** C. Richards

**Affiliations:** Joliet, Ill.


### EXAMINER.

Vol. XXXIII.—SEPTEMBER, 1876.—No. 9.

### Original Communications.

### OVARIOTOMY IN A PATIENT SIXTY-FIVE YEARS OF AGE, WITH COMPLETE RECOVERY.

By C. RICHARDS, M.D., Joliet, Ill.

Mrs. K., aged sixty-five years, in the early part of 1872, discovered a tumor about the size of a small orange in the left inguinal region. It enlarged slowly until August, 1874, when she could not longer endure the severe pressure upon the abdominal viscera. In August, 1874, she was tapped, and a thin whitish colored fluid was evacuated, weighing thirty-four pounds. In May, 1875, she was again tapped; again in August, 1875; again on the 15th of February, 1876, when sixteen pounds of fluid were removed; again March 4th, 1876, when twenty-three pounds were removed; again March 17th, 1876, when fifteen pounds were removed. On April 6th, the tumor and fluid were removed together, and weighed about twenty-three pounds. It will be thus seen that the time between each tapping gradually decreased. The patient was of lymphatic temperament, and prior to a short time before the operation had enjoyed good health. But after the last tapping she rapidly failed, both in strength and flesh; the stomach becoming very irritable. The operation was performed at Plainfield, Ill., April 6th, 1876, in the presence, and with the assistance of the following medical gentlemen: Drs. R. J. Curtiss, F. C. Mitchell, and Wm. M. Richards, of Joliet, and Drs. D. W. Jump and A. J. Perkins, of Plainfield, Ill. The patient was brought fully under the influence of ether, and the bladder was then emptied of its contents. A small incision, about three inches in length, was made in the linea alba. The finger was then introduced, and what adhesions could be reached were broken up. The sound was next introduced for the purpose of exploration, and several slight adhesions were discovered at different points over the abdomen, which were easily removed. One somewhat more extensive and firm was found in the right inguinal region. This required separating by the fingers. The incision was now enlarged to about nine inches, and the tumor found to be multilocular, chiefly made up of two very large cysts and a great many smaller ones. The larger cysts were now tapped with a large trocar, and the fluid, which was of a yellow color and somewhat viscid, rapidly drawn off. Not much solid substance was encountered. The sac was then seized and drawn out. The pedicle, which was of medium length, was then tied firmly with a strong silken ligature. The clamp placed on the pedicle below the ligature and the sac cut away. The incision was then closed by deep silver sutures, inserted so as to include the skin and peritoneum, and by superficial silk ligatures, inserted so as to approximate the cutaneous edges. Adhesive straps were applied, and a cotton compress over the wound was held in position by a broad flannel bandage. The pulse being weak, an injection of brandy and water was given *per rectum* and the patient put to bed. The temperature of the room during the operation was kept at about 80°·Fahr. The following is a correct record of the symptoms and treatment:

April 6, 1876, 12:25 P. M., pulse 100, temperature normal; 1:25 P. M., pulse 117, temperature normal; 4 P. M., pulse 98, temperature 98°; 7 P. M., pulse 99, temperature 98$\frac{4}{5}$°; 12 P. M., pulse 102, temperature 98°. *Treatment*—2:15 P. M., morphine, gr. $\frac{1}{4}$, atropia sulph., gr. $\frac{1}{60}$, hypodermically; 5 P. M., injection of beef tea and brandy; 5:30 P.M., morphine, gr. $\frac{1}{4}$, and atropia, gr. $\frac{1}{60}$, hypod.; 9:40 P.M., mophine, gr. $\frac{1}{4}$, and atropia gr. $\frac{1}{60}$, hypod.

April 7, 1876, 2 A.M., pulse 100, temperature 98$\frac{2}{5}$°; 6 A.M., pulse excited, temperature 98$\frac{3}{5}$°; 12 A.M., pulse 98, temperature 98$\frac{3}{5}$°; 8 P.M., pulse 100, temperature 98$\frac{4}{5}$°; 10 P.M., pulse 100, temperature 99°. *Treatment*—12:30 A.M., beef tea, o. j., brandy ℥ j., by injection; 4:30, morphine and atropia; 6:30, beef tea, brandy and milk; 10 A.M., action of heart being weak, two drops of tinct. digitalis were given and continued every two hours until the heart's action became strong and steady. 10:30 A.M. beef tea, o. j., brandy ℥ jss., quinia, gr. x. M. By injection. 12:30 P.M., morphine, gr. $\frac{1}{4}$, atropia, gr. $\frac{1}{60}$, hypodermically; 4:30, morphia and atropia, as above; 5, beef tea, brandy and milk; 9 A.M., morphia and atropia; 10, beef tea, brandy and milk.

April 8. Symptoms: 12:45 A.M., pulse 106, temperature 99$\frac{4}{5}$°; vomited about a pint of dark colored matter; 4 A.M., pulse 114, temperature 100$\frac{1}{5}$°; 11:30 A.M., pulse 106, temperature 99°; vomits; 12 P.M., bowels moved; 12:30, vomiting of mucus with bile; 6, pulse 110, temperature 100°; vomited fetid matter, tinged with bile; bowels moved slightly and without pain; 7:40 P.M., pulse 108, temperature 99°; 9:45, bowels moved without pain; 10:30, pulse 100, temperature 98$\frac{1}{2}$°. Up to this time patient has had but little disturbed sleep; at times delirious; tongue dry with a brown center. Bismuth gr. v., pepsine gr. x., every four hours. 1 P.M., beef tea, brandy, milk and quinine, by injection; 2:30, morphine and atropia; 6, beef, milk and brandy; 9, beef, milk and brandy; 11:30, morphine and atropia.

April 9. 1 A.M., beef tea, o. ss., milk o. j., brandy ℥ j.; 6, morphine and atropia hypodermically; 6:30, beef tea, brandy and milk; 10, morphia and atropia; 10:45, beef tea, brandy and milk; 3 P.M., stomach irritable; milk ℥ j., aqua calcis ℥ ss. every hour; 5, morphine and atropia; 10, morphine and atropia, hypodermically, brandy and water in connection with lime-water and milk; stomach less irritable.

April 10. 1 A.M., milk in large quantities with half ounces lime-water; 4, lime-water, milk and brandy; no nausea. *Symptoms*—3 A. M., tongue moist, pulse 90, temperature 98°; has rested well during the night. 6 A. M., pulse 90, temperature 98°; milk and aqua calcis; desires food. 1 P. M., beef, bread and butter retained well, temperature normal, pulse 99.

April 11. 1 A. M., pulse 96, temperature 98°; did not rest well before this time; morphia and atropia were given with relief of symptoms. 8 A. M., pulse 90; temperature 98°. Egg and toast for breakfast; relished her food. 1:30 P. M., pulse 96, temperature 98°. 9:30, pulse 88, temperature 98°.

April 12. 7 A. M., pulse 90, temperature 98°. Rested well during the night. 2:30 P. M., pulse 100; temperature 99°.

April 13. 8:40 A. M., pulse 94; temperature 98°. Eggs, toast and tea; appetite good. 8 P. M., pulse 90; temperature $98\frac{1}{2}$°.

April 14. Pulse 86; temperature 98°; rested well during the night. At 9 P. M. temperature 94°; pulse 90. Soreness about pedicle.

April 15. Pulse 84; temperature 98°. Slept well during the night; appetite good. Pedicle came off on the 16th, and patient went on to a rapid recovery. At present, June 2, 1876, she is enjoying as good health as could be expected. In the treatment of the pedicle little more was done than keeping the parts clean, applying carbolized glycerine at the time of each dressing. Drs. Jump and Richards remained with the patient from the time of the operation to the separation of the pedicle, which took place on the tenth day after the operation—for the success of which I am largely indebted to all who assisted me.

